# Efficacy of vaccine-induced Vif-specific CTL responses against SIVmac239 infection: implications for antigen design in AIDS vaccines

**DOI:** 10.1186/1742-4690-9-S2-P26

**Published:** 2012-09-13

**Authors:** N Iwamoto, N Takahashi, T Nomura, H Yamamoto, T Matano

**Affiliations:** 1 National Institute of Infectious Diseases, & IMS, University of Tokyo, Tokyo, Japan; 2 National Institute of Infectious Diseases, Tokyo, Japan

## Background

Optimization of antigens as well as delivery system is crucial for development of an effective T-cell based AIDS vaccine. Our recent results suggested higher anti-viral efficacy of Vif- and Nef-specific CTLs as well as Gag-specific ones (JEM 199:1709, 2004; AIDS 24:2777, 2010). Here, we examined efficacy of Gag-specific or Vif/Nef-specific CTL induction by vaccination against SIV infection.

## Methods

All 17 animals used in this study were Burmese rhesus macaques sharing MHC-I haplotype 90-010-Ie, which mostly show typical AIDS progression after SIVmac239 challenge (geometric means of setpoint plasma viral loads: 10^5 copies/ml; mean survival periods: 2 years). These animals were divided into three groups consisting of unvaccinated (n = 6), Gag-vaccinated (n = 5), and Vif/Nef-vaccinated (n = 6); the latter two were subjected to DNA-prime/Sendai virus vector-boost vaccination. We compared these three groups after an intravenous SIVmac239 challenge.

## Results

After challenge, 3 out of 5 Gag-vaccinated and 3 out of 6 Vif/Nef-vaccinated animals controlled SIV replication. The SIV control was associated with Gag-specific CTL responses in the former and Vif-specific CTL responses in the latter.

## Conclusion

This is the first report indicating efficacy of vaccine-induced Vif-specific CTL responses against SIV replication. Our results imply that not only Gag but also Vif may be a promising antigen for T-cell based AIDS vaccines.

